# A Novel Quinolone JH62 (E-2-(Tridec-4-en-1-yl)-quinolin-4(1H)-one) from *Pseudomonas aeruginosa* Exhibits Potent Anticancer Activity

**DOI:** 10.3390/microorganisms14010078

**Published:** 2025-12-30

**Authors:** Qunyi Chen, Jianhe Wang, Xiaoyan Wu, Lantu Xiong, Lianhui Zhang, Zining Cui

**Affiliations:** Integrative Microbiology Research Centre, South China Agricultural University, Guangzhou 510642, China; chenqy@stu.scau.edu.cn (Q.C.); jhwang@scau.edu.cn (J.W.); wuxy@stu.scau.edu.cn (X.W.); xionglt0618@stu.scau.edu.cn (L.X.)

**Keywords:** cancer therapy, *Pseudomonas aeruginosa*, A549 lung cancer cells, anticancer compounds, quinolone

## Abstract

Cancer remains a leading cause of mortality worldwide, and new chemical leads are essential for developing potent anticancer therapies. Evidence suggests that *Pseudomonas aeruginosa* (*Pa*) may suppress tumorigenesis, although the underlying mechanisms remain largely unclear. This study characterized a novel small molecule quinolone, JH62 (E-2-(tridec-4-en-1-yl)-quinolin-4(1H)-one, C_22_H_31_NO), from *Pa*. JH62 exhibited broad-spectrum anticancer activity, inhibiting the proliferation of A549 lung cancer cells in a time- and dose-dependent manner with an IC_50_ of 15 μM, while showed low cytotoxicity toward normal cells. In xenograft mice model, treatment with JH62 (10 mg/kg) reduced tumor weight and volume by 73% and 79%, respectively. Mechanistically, treatment with JH62 induced structural and functional disruption of mitochondria in cancer cells, triggered autophagic cell death, and did not cause DNA damage. Genetic analysis confirmed that JH62 biosynthesis depends on the *pqsABCDE* gene cluster and that JH62 positively regulates its own production. ADMET profiling further indicated promising drug-like properties for future development. These findings establish JH62 as a promising anticancer lead compound derived from microbial metabolism.

## 1. Introduction

The complex interplay between microorganisms and human diseases has drawn increasing scientific attention in recent years. Microorganisms can disrupt host immune function and contribute to physiological and biochemical dysregulation, thereby accelerating disease onset and progression [1]. For instance, *Helicobacter pylori* infection not only causes gastroenteritis and gastroduodenal ulcers, but also promotes the onset of gastric cancer [2]. Gut bacterial disorder causes intestinal ailments, and is associated with diabetes, chronic heart diseases, cancers and obesity [3]. Intriguingly, certain bacteria can also exert protective effects. Historical reports dating back over a century describe the use of *Streptococcus* cultures to inhibit tumor growth and improve patient outcomes [4]. The abundance of *Akkermansia muciniphila* is significantly higher in healthy individuals compared to patients with obesity, diabetes, cardiovascular disease, and nonalcoholic fatty liver disease [5]. *Bifidobacterium* spp. have been used for the treatment of acute diarrhea, necrotizing enterocolitis, and allergies [6]. Modulation of the gut microbiome could potentially alleviate symptoms of Alzheimer’s disease [7]. Apparently, understanding the underlying mechanisms by which microorganisms influence the progress of diseases may shed light and provide clues for disease prevention and control.

Due to the elevated incidence and mortality rates, cancer stands as one of the most lethal human diseases, posing a significant public health challenge on a global scale [8]. Clinically, conventional cancer therapies, including chemotherapy, radiotherapy, and surgery, can effectively suppress cancer progression to a certain extent. However, these methods are limited by their side effects and recurrence rates [9]. Therefore, the development of novel, effective therapies to complement or replace conventional treatments offers promising therapeutic options for millions of cancer patients [10]. Among emerging approaches, microbe-mediated anticancer therapy represents a promising strategy, with microbial metabolites serving as a particularly attractive resource [11]. For instance, Bleomycin, a glycopeptide produced by *Streptoalloteichus hindustanus*, has been used for squamous cell carcinomas, Hodgkin’s lymphomas, and testis tumors [12]. *Streptomyces spinoverrucosus* produces galvaquinone B, which exhibited cytotoxicity against non-small-cell lung cancer (NSCLC) cell lines Calu-3 and H2887 [13].

As a versatile opportunistic pathogen, *Pseudomonas aeruginosa* (*Pa*) is a primary source of hospital-acquired infections, associated with high morbidity and mortality in immunodeficient and cystic fibrosis (CF) patients [14]. Intriguingly, epidemiological studies indicate that CF patients display a lower incidence of melanoma and breast cancer [15,16]. Experimentally, the supernatant of *Pa* could induce apoptosis in tumor-derived J774A.1 macrophages [17], and a *Pa* clinical isolate was shown to trigger tumor cell necrosis via the TLR4-RIP3-MLKL pathway [18]. These findings suggest that *Pa* may possess the ability to suppress tumorigenesis and tumor progression. Accordingly, a phase II clinical trial reported that the combination of inactivated *Pa* with Capecitabine improved the survival of metastatic breast cancer patients from 16.4 months to 25.4 months [19]. A protein toxin exotoxin A produced by *Pa* showed potent anticancer activity both in vitro and in vivo, which inhibited protein synthesis through ADP-ribosylation of eukaryotic elongation factor 2 [20]. Azurin, a periplasmic copper-containing redox protein secreted by *Pa*, exhibited strong cytotoxicity towards melanoma and breast cancer cells [21]. However, exotoxin A and azurin are both proteins with large molecular weights, 66 kDa and 14 kDa, respectively, which may constrain their clinical applications.

*Pa* possesses a relatively large genome and is capable of producing a broad-spectrum of secondary metabolites [22]. The key metabolites of *Pa* are summarized in Table 1. The biological activity of many secondary metabolites in *Pa* remains largely uncharacterized, representing an important and unexplored resource for identifying new potential anticancer compounds [23]. Among diverse secondary metabolites, quinolone compounds produced by *Pa* have garnered sustained attention because of their remarkable structural diversity and broad biological activities. The isolation of quinolones from *Pa* dates back to the 1950s, with the initial identification of several antibiotic molecules such as HQNO, NQNO, HHQ, and NHQ [24]. A pivotal advance came in 1999 with the discovery by Pesci et al. [25] of the quorum sensing (QS) signal molecule PQS, which established quinolones as key regulators of bacterial communication. Advances in analytical technologies have since led to the characterization of numerous additional quinolone derivatives (e.g., UQNO, DHQ, AQNO), and it is now estimated that *Pa* produces over 50 structurally distinct quinolone compounds [26,27]. Beyond their role in QS, these molecules exhibit diverse bioactivities, including the modulation of virulence factor expression and direct interactions with host cells [28]. Notably, during infection, *Pa*-derived quinolones such as PQS, HHQ, and HQNO can interfere with eukaryotic signaling pathways (e.g., NF-κB, HIF-1), thereby attenuating host innate immune responses [29]. These findings underscore that *Pa*-derived quinolones function not only as bacterial signaling molecules but also as bioactive agents capable of modulating eukaryotic cell physiology. Despite this growing understanding, the potential effects of *Pa*-derived quinolones specifically on cancer cells remain largely unexplored, presenting a promising avenue for anticancer drug discovery.

In this study, we isolated and identified a novel compound, JH62, from *Pa* PAO1, which has promising potential as a targeted and low toxicity therapy agent against cancer. The results demonstrate that JH62 inhibits the growth and colony formation of A549 lung cancer cells in a time- and dose-dependent manner, with an IC_50_ value of 15 μM. JH62 exhibits broad-spectrum anticancer activity by suppressing the proliferation of various cancer cell lines while showing low toxicity toward normal somatic cells. In xenograft mice, JH62 treatment significantly reduced both tumor volume and weight. Structural analysis via nuclear magnetic resonance (NMR) spectroscopy revealed JH62 to be E-2-(tridec-4-en-1-yl)-quinolin-4(1H)-one, with the chemical formula C_22_H_31_NO. Subsequent research revealed that JH62 induces mitochondrial functional and structural disruption in A549 lung cancer cells. Unlike the chemotherapeutic agent Doxorubicin (DOX), JH62 does not cause DNA damage. Analysis of the cell death pattern in A549 cells suggest the involvement of autophagy. We further confirmed that JH62 biosynthesis depends on the *pqsABCDE* gene cluster and that JH62 positively regulates its own production via feedback upregulation of this cluster. Favorable drug-like properties of JH62 were further supported by ADMET profiling. Together, these findings provide a scientific foundation for the development of JH62 as a promising anticancer lead compound.

## 2. Materials and Methods

### 2.1. Cell Lines

Different cells were utilized as follows: MEF: murine embryonic fibroblast (RRID: CVCL_L690); AML12: mouse liver cell lines (RRID: CVCL_0140); WI-38: human lung fibroblast cell lines (RRID: CVCL_0579); THLE-2: human liver epithelial cell lines (RRID: CVCL_3803); A549: human lung cancer cell lines (RRID: CVCL_0023); HEPG2: human liver cancer cell lines (RRID: CVCL_0027); HEP3B: human hepatoma cell lines (RRID: CVCL_0326); BxPC3: human pancreatic cancer cell lines (RRID: CVCL_0186); U87: human primary glioblastoma cell lines (RRID: CVCL_0022); DU145: human prostate cancer cell lines (RRID: CVCL_0105); MCF7: human breast cancer cell lines (RRID: CVCL_0031); HeLa: human cervical cancer cell lines (RRID: CVCL_0030); HCT116: human colon cancer cell lines (RRID: CVCL_0291). Cells were maintained in Dulbecco’s modified Eagle’s medium (DMEM, Gibco) supplemented with L-glutamine (Lonza) (2 mM), 10% fetal bovine serum (FBS, Hyclone), 1 × non-essential amino acids, and sodium pyruvate (1 mM). THLE-2 cells were maintained in THLE-2 Cell Complete Medium (Zhong Qiao Xin Zhou, Shanghai, China). Cells were incubated at 37 °C in a humidified incubator with 5% CO_2_. A549 cells were previously stored in the lab and other cell lines were a gift from Haishan Wang’s lab (Institute of Molecular and Cell Biology, Singapore). All cell lines were confirmed to be contamination-free.

### 2.2. Pa Strains

Different *Pa* strains were utilized as follows: *Pa* PAO1 was used as a parental strain for compound isolation, generation of mutants, and complementary strains. Other *Pa* strains (PAO1-1, PAO1-2, PAO1-3, PAO1-4, PAO1-5, CF4, PF4, NTU, PA14) were also from laboratory collections. All *Pa* strains were routinely grown at 37 °C in Luria-Bertani broth (LB).

### 2.3. Mice

The BALB/c nude mice were purchased from the Guangdong Medical Laboratory Animal Center (Guangzhou, China).

### 2.4. Reagents

DOX in aqueous solution was purchased from Sigma (Sigma-Aldrich, Singapore). JH62 was synthesized by U-CHEMO Holding Co., Limited (Hong Kong, China), based on reported procedures [51]. The antibodies against LC3B were purchased from Abcam (Cambridge, UK).

### 2.5. Preparation of Bacterial Supernatants and Purification of Inhibitory Compound

Bacterial culture supernatants and inhibitory compounds were prepared and purified following previously described procedures with slight modifications [52]. *Pa* PAO1 was grown overnight in LB as a starter culture. Bacterial cultures were adjusted to an OD600 of 1.0 and then inoculated (1% vol/vol) into fresh LB medium contained in 2 L glass conical flasks. Cultures were shaken at 200 rpm and 37 °C. Bacterial cultures were grown for 48 h. The total culture volume (20 L) was centrifuged and the bacterial supernatants were acidified to pH 3.5 by adding HCl, then extracted with an equal volume of ethyl acetate. The combined organic phases were evaporated to dryness, and the residue was dissolved in methanol (10 mL). The crude extracts were purified by HPLC using a C18 reverse-phase column (4.6 × 250 mm, Waters Corporation, Milford, MA, USA), eluted with an acetonitrile–water gradient (80:20 vol/vol) at a flow rate of 1 mL/min. Absorbance was monitored using a Waters 2487 Dual λ Absorbance Detector (Waters Corporation, Milford, MA, USA). Peaks corresponding to compounds of interest were collected and assayed for cytotoxicity against A549 cells.

### 2.6. Chemical Structural Analysis

Chemical structural analysis was performed by nuclear magnetic resonance (NMR) [53]. NMR spectra were obtained using a Bruker Avance instrument (^1^H and ^13^C NMR at 500 and 126 MHz; ^1^H-^13^C NMR at 125 MHz), with chemical shifts referenced to the solvent peak. The product was further confirmed by high resolution mass spectrometry (HRMS) using negative ion mode electrospray ionization (MicrOTOF-QII, Bruker Daltonics, Bremen, Germany). Additionally, electron impact (EI) mass spectra were obtained at 20–40 eV.

### 2.7. Cell Viability

Cell viability was assessed using the WST-1 assay, based on a previously reported method with slight modifications [54]. Briefly, cells were seeded at 1 × 10^4^ cells per well in 96-well plates and then treated with different doses of JH62 or DOX for 0–72 h. The cells used included non-cancer cells (MEF, AML12, WI-38, and THLE-2) and cancer cells (A549, HEPG2, HEP3B, BxPC3, U87, DU145, MCF7, HeLa, and HCT116). The WST-1 reagent (Roche, Shanghai, China) was added to each well (10% vol/vol final concentration), followed by incubation for 3 h at 37 °C. Absorbance at OD450 was measured using a microplate reader (Synergy H1; BioTek Instruments, Winooski, VT, USA).

### 2.8. Drug Combination Study

The synergistic effect of JH62 and DOX was evaluated using a matrix-based combination study in 96-well plates, as previously described with minor modifications [55]. Briefly, cells were seeded at 1 × 10^4^ cells per well in 96-well plates. The cells used included non-cancer cells (MEF, AML12, WI-38, and THLE-2) and cancer cells (A549, HEPG2, HEP3B, BxPC3, U87, DU145, MCF7, HeLa, and HCT116). Serial dilutions of JH62 and DOX were prepared based on their predetermined individual IC_50_ values. A concentration matrix was generated by combining each concentration of JH62 with each concentration of DOX, and the mixtures were applied to the cells. Cells treated with single agents or vehicle (DMSO) served as controls. After 24–48 h of incubation, cell viability was determined using the WST-1 assay according to the manufacturer’s protocol (Roche), and absorbance was measured at OD450 using a microplate reader.

Drug interaction was analyzed using the SynergyFinder web tool (https://synergyfinder.fimm.fi/, accessed on 20 December 2025). The dose–response matrices were uploaded, and synergy scores were calculated using the Zero Interaction Potency (ZIP) model. According to the model’s criteria, a ZIP synergy score less than −10 indicates antagonism, a score between −10 and 10 indicates an additive effect, and a score greater than 10 indicates synergy.

### 2.9. Tumorigenicity Assay

Tumorigenicity assays were performed using BALB/c nude mice, adapted from previously described protocols with slight modifications [56]. To establish the xenograft cancer model, cancer cells (1 × 10^6^) were subcutaneously injected into the right dorsal side of 6-week-old male BALB/c nude mice to induce tumor formation. The mice were randomly divided into 2 groups (*n* = 15). One group served as the control, and the other groups were treated with JH62 at doses of 10 mg/kg, via intraperitoneal injection twice a week. The mice in the control group were administered an equal volume of DMSO as the solvent control. The tumor sizes were measured weekly for three weeks. After that, the mice were sacrificed, and the tumors were isolated and weighed. All animal studies were approved by the Ethics Committee of South China Agricultural University.

### 2.10. Mitochondria JC-1 and MitoTracker Staining

Mitochondrial membrane potential and mitochondria localization were assessed by JC-1 and MitoTracker staining based on previously published methods [57]. A549 cells were cultured at 37 °C and 5% CO_2_ in Dulbecco’s modified Eagle’s medium (DMEM), supplemented with 10% heat-inactivated FBS (Thermo Fisher Scientific, Waltham, MA, USA) and L-Glutamine (Thermo Fisher Scientific, Waltham, MA, USA) (2 mM). An aliquot of 1 mL of JH62 (25 μM) solution was added to the cell cultures. For JC-1 staining, cells grown in 24-well plates were treated as indicated for 12 h, washed with PBS, incubated with JC-1 solution according to the manufacturer’s instructions for 10 min at 37 °C, and then washed again. Cells were washed with dilution buffer, and photos were taken using a microscope. For MitoTracker staining, cells grown on 12 mm diameter round glass coverslips in 24-well plates were incubated with MitoTracker Red diluted 1:2000 or 1:20,000 from a stock solution (1 mM) in culture medium for 45 min at 37 °C. Cells were then washed with PBS and fixed with ice-cold 3.2% paraformaldehyde in PBS for 20 min at room temperature. After washing, coverslips were mounted on glass slides using an appropriate mounting medium. Photos were taken using a microscope.

### 2.11. Chromosome Staining Experiment

Metaphase chromosome spreads were prepared from A549 cells. Briefly, A549 cells were cultured. After treatment with JH62 or DOX, cells were incubated with Colcemid (Gibco BRL; 0.2 μg/mL final concentration) for 2 h to arrest cells in metaphase. Cells were then subjected to hypotonic treatment in KCl (0.075 M) at 37 °C for 5 min, and fixed by repeated washing with ice-cold freshly prepared 3:1 (vol/vol) methanol:acetic acid. Chromosome spreads were prepared on premarked glass slides using the “drop-cryo” protocol [58]. The chromosome suspension was dropped onto a slide. This was followed by adding three drops of 45% acetic acid and placing a coverslip over the suspension. The slide was then inverted and placed on dry ice for 15 min. The spreads were postfixed in 2.5% glutaraldehyde in cacodylate buffer (pH 7.4, 75 mM) for 15 min and washed with cacodylate buffer (0.1 M). The chromosomes were stained with Vista Green DNA dye (Cell Biolabs, Singapore). After staining, the preparations were washed in water for 5, 10, and 15 min, then dehydrated using a series of ethanol solutions as follows: 5 min in a 70% solution, 10 min in an 85% solution, and 15 min in 100% ethanol. Finally, slides were mounted with a permanent mounting medium, sealed, and analyzed by microscopy.

### 2.12. DNA Fragmentation Analysis

Genomic DNA fragmentation was analyzed by agarose gel electrophoresis using previously described methods [59]. A549 cells were seeded in 6 cm tissue culture dishes and cultured overnight. Before treatment with JH62 or DOX, the cells were cultured in DMEM lacking FBS for 16 h. After treatment, the cells were then washed three times with PBS and genomic DNA was extracted using a MasterPure™ DNA Purification Kit (Lucigen Corporation, Middleton, WI, USA). The DNA pellet was airdried and resuspended in Tris-HCl (pH 8.0, 10 mM). DNA samples (2.5 μg) were separated by 1% agarose gel electrophoresis in TAE buffer containing ethidium bromide.

### 2.13. Comet Assay

Residual DNA damage was detected by alkaline single cell gel electrophoresis (Comet Assay) using the Trevigen Comet Assay Kit (Trevigen, Gaithersburg, MD, USA) following the manufacturer’s protocol with slight modifications [60]. Briefly, cells were grown in 6-well plates to approximately 30% confluence and treated with DOX or JH62 for 6 h. The treated cells were trypsinized and pelleted by centrifugation. The cell pellets were washed and resuspended in 2 mL of ice-cold PBS. The cell suspension was mixed 1:10 (vol/vol) with low-melting point agar at 42 °C and immediately pipetted onto a CometSlide™. Slides were incubated at 4 °C in the dark for 30 min, immersed in chilled lysis solution and incubated again at 4 °C for 30 min. Slides were placed in a horizontal electrophoresis chamber and electrophoresed with buffer NaOH (0.3 M) and EDTA (1 mM) at 1 V/cm for 15 min. Samples were dried and stained with Vista Green DNA dye (Cell Biolabs, Singapore). Comet tails were imaged using a model DM 2500 fluorescent microscope (Leica, Wetzlar, Germany) and quantified by Komet 5.5 software (Andor Technology, Belfast, UK). A minimum of 50 cells were scored per treatment. All experiments were performed independently in triplicate.

### 2.14. JH62 Extraction and Quantitative Analysis

The extraction and quantitative analysis of JH62 were performed based on previously published methods, with minor modifications [61]. Corresponding *Pa* PAO1 and mutant strains were grown in LB medium at 37 °C overnight. An aliquot of 50 µL of bacterial culture (OD600 of 1.0) was diluted to 5 mL fresh LB medium in 50 mL tubes. The tubes were shaken at 37 °C. At the desired time point, culture supernatant was separated by centrifuging at 13,000 rpm for 5 min. Same volume of ethyl acetate with 0.1% acetic acid was added into the supernatant samples, and the samples were rapidly vortex mixed for 5 min. The organic phases were separated by centrifuging at 1000 rpm for 5 min, and transferred to new tubes. Acidified ethyl acetate was added into the sample, and the extraction was repeated again. All of the organic phases were collected together and evaporated to dryness. An aliquot of 200 µL of methanol was added to dissolve residues. The resulting solution was used for quantitative analysis of JH62.

Intracellular JH62 extraction was carried out by resuspending the cell pellet with 5 mL of fresh LB medium, followed by sonication disruption and centrifugation at 13,000 rpm for 5 min to obtain the disrupted supernatant. The same extraction described above was performed on the disrupted supernatant.

Liquid chromatography-mass spectrometry (LC-MS) was used for the quantitative analysis of JH62. Signal mass scans were set for JH62 at 326.2478 *m*/*z*. Data analysis was performed using Thermo Xcalibur software version 4.1.5 (Thermo Fisher Scientific, Waltham, MA, USA).

### 2.15. Construction of Pa Deletion Mutant and Complemented Strains

The construction of *Pa* deletion mutants and complemented strains was performed as previously described [62]. For the generation of in-frame deletion mutants, the gene replacement vector pK18mobsacB derivatives, which were ligated with the 500bp upstream and 500 bp downstream sequences of target genes, were transformed into corresponding parental strains with the helper vector pRK2013 by triparental mating. Desired mutants were counter-selected on LB plates containing 10% sucrose and verified by PCR and DNA sequencing. For the construction of complementation and overexpression strains, the promoter and ORF region of target genes were cloned into pBBR1-MCS5 that had been digested, and then transformed into corresponding strains by triparental mating. All of the resultant constructs were confirmed by PCR analysis and DNA sequencing.

### 2.16. ADMET (Absorption, Distribution, Metabolism, Excretion and Toxicity) Analysis

The ADMET analysis was conducted using the online platforms SwissADME (http://www.swissadme.ch/, accessed on 10 May 2025) and ADMETlab 3.0 (https://admetlab3.scbdd.com/, accessed on 10 May 2025), following previously published protocols [63].

### 2.17. Data Analysis

All data are presented as the mean ± SD (Standard deviation) of at least three independent experiments. Statistical analyses were performed using GraphPad Prism software version 9.5. Comparisons between two groups were conducted using the Student’s *t*-test, while comparisons among multiple groups were analyzed by one-way analysis of variance (ANOVA). Statistical significance was defined as *p* < 0.05 (ns, not significant; * *p* < 0.05; ** *p* < 0.01; *** *p* < 0.001; **** *p* < 0.0001).

### 2.18. Ethics Statement

The animal study was reviewed and approved by the Ethics Committee of South China Agricultural University.

## 3. Results

### 3.1. A Novel Quinolone Compound JH62 Isolated from Pa Inhibits Cancer Cell Viability

**Figure 1:**
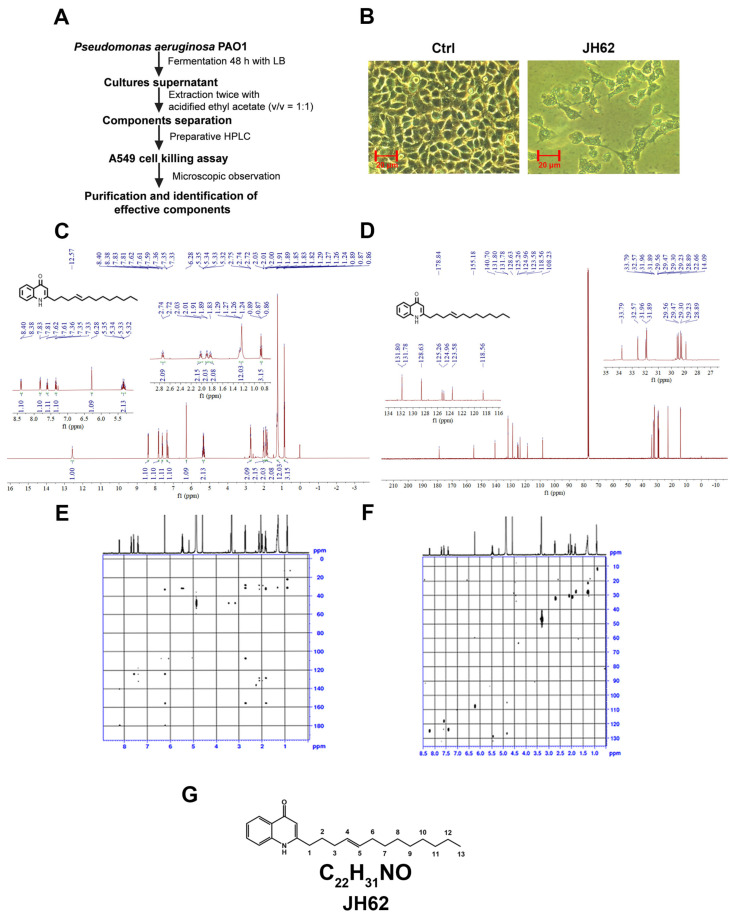
Isolation and structural characterization of JH62, an anticancer compound from *Pa*. (**A**) Flowchart for extraction and isolation of anticancer compounds from *Pa* PAO1. (**B**) Microscopic analysis of the effect of JH62 fraction on the growth of A549 lung cancer cells. (**C**) ^1^H NMR spectrum of JH62. (**D**) ^13^C NMR spectrum of JH62. (**E**) HMBC (^1^H−^13^C Heteronuclear Multiple Bond Correlation) spectrum of JH62. (**F**) HMQC (^1^H-^13^C Heteronuclear Multiple Quantum Coherence) spectrum of JH62. (**G**) Chemical structure and molecular formula of the compound JH62.

Prompted by the finding that *Pa* is capable of inducing apoptosis and necrosis in several cancer cell lines [64], we screened for small molecules with anticancer activity using A549 lung cancer cells, following the procedure outlined in Figure 1A. *Pa* PAO1 was cultured in LB medium for 48 h prior to extraction using acidified ethyl acetate. Crude extracts were separated through preparative HPLC, and 15 mL fractions were collected at 5 min intervals. The fractions were appropriately diluted and added to A549 cells growing in a 96-well plate. Cell viability was monitored daily by microscopy to identify fractions exhibiting cytotoxic activity. One active fraction, designated JH62, consistently inhibited the growth and proliferation of A549 cells. Microscopic examination showed that A549 cells treated with the JH62 crude fraction showed reduced adherence and failed to form a confluent monolayer (Figure 1B).

To analyze the chemical structure, the crude fraction containing JH62 was subjected to further separation and purification to obtain a relatively pure JH62 compound (Figure S1). The chemical structure of JH62 was determined by 1D NMR (^1^H and ^13^C) and 2D NMR (HMBC and HMQC) analysis, with key correlations in the 2D NMR spectra confirming the overall structure and the attachment of the side chain (Figure 1C–F and Figure S2). JH62 was identified as E-2-(tridec-4-en-1-yl)-quinolin-4(1H)-one, a quinolone derivative featuring a 13-carbon unsaturated alkyl side chain. The chemical formula of JH62 is C_22_H_31_NO, with a molecular weight of 325.48 Da, identifying it as a quinolone compound that has not previously been reported in *Pa* (Figure 1G). For subsequent experiments, JH62 was custom-synthesized by U-CHEMO Holding Co., Limited (Hong Kong, China) following a reported synthetic procedure [51], which describes the stepwise construction of the quinolone core and subsequent installation of the alkyl side chain. The synthesized JH62 compound exhibited identical UV spectral characteristics to the JH62 molecule purified from *Pa*, and its molecular weight and purity were confirmed by LC-MS analysis (Figure S3).

### 3.2. JH62 Induces A549 Cell Death and Inhibits Tumor Growth

**Figure 2:**
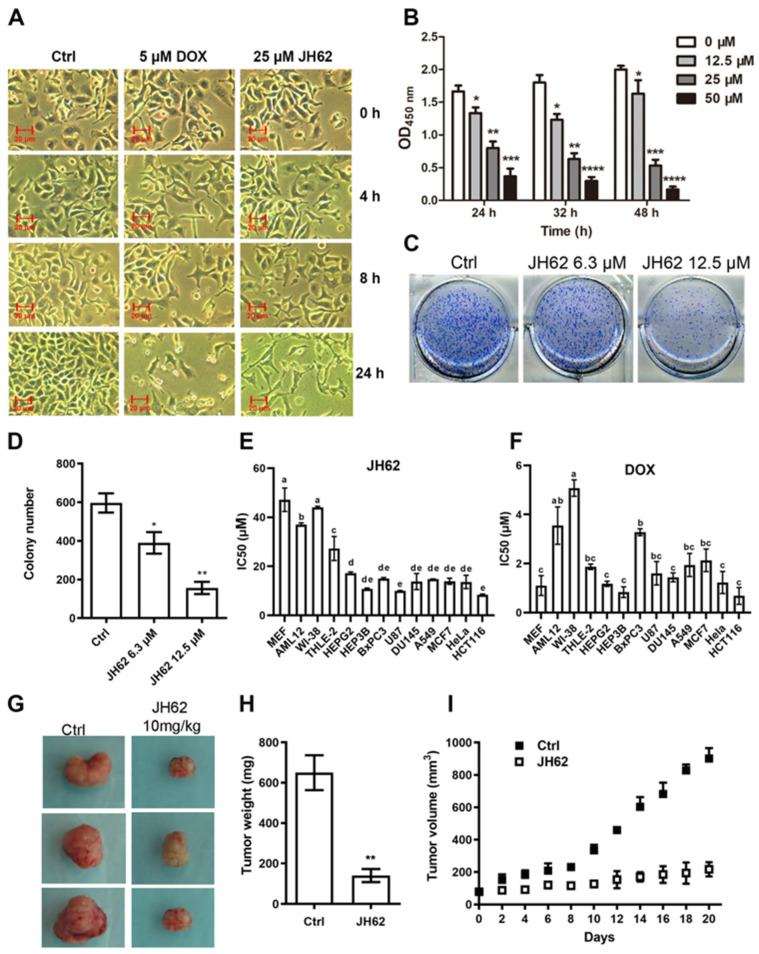
JH62 induces cancer cell death and inhibits tumor growth. (**A**,**B**) JH62 inhibits the growth of A549 lung cancer cells. Microscopic images of A549 cells treated with JH62 or DOX. Cells were treated with 25 μM JH62 or 5 μM DOX for 0, 4, 8, and 24 h. Ctrl represents the solvent control group treated with an equal volume of DMSO (**A**); Effect of JH62 on the viability of A549 cells. Cells were treated with 12.5, 25, and 50 μM JH62 for 24, 32, and 48 h. The 0 μM group represents the solvent control (equal volume of DMSO) (**B**). Colony formation (**C**) and colony number quantification (**D**) of A549 cells with 6.3 μM or 12.5 μM JH62 treatment. IC_50_ values for JH62 (**E**) and DOX (**F**) against different cell lines. MEF, mouse embryonic fibroblast cell lines. AML12, mouse liver cell lines. WI-38, human lung fibroblast cell lines. THLE-2: human liver epithelial cell lines. HEPG2, human liver cancer cell lines. HEP3B, human hepatoma cell lines. BxPC3, human pancreatic cancer cell lines. U87, human primary glioblastoma cell lines. DU145, human prostate cancer cell lines. A549, alveolar adenocarcinoma cell lines. MCF7, human breast cancer cell lines. Hela, human cervical cancer cell lines. HCT116, human colon cancer cell lines. The appearance (**G**), weight (**H**) and volume (**I**) of tumors from A549 injected xenograft mouse model with 10 mg/kg JH62 treatment. *n* = 15. Ctrl, Dimethyl sulfoxide (DMSO) as solvent controls. Statistical analysis was performed using one way ANOVA (**B**,**D**–**F**) or Student’s *t*-test (**H**). * *p* < 0.05; ** *p* < 0.01; *** *p* < 0.001; **** *p* < 0.0001. Bars in (**E**,**F**) sharing the same letter are not significantly different, whereas bars with different letters indicate statistically significant differences (*p* < 0.05). Error bars indicated standard deviations.

Further experiments were performed to understand the characteristics of JH62-induced cancer cell death. A549 cells were first treated with 25 μM of synthesized JH62 for 0, 4, 8, and 24 h. As shown in Figure 2A and Figure S4, prolonged exposure to JH62 markedly attenuated proliferative capacity, leading to reduced cell adhesion, widened intercellular gaps, and failure to form confluent monolayers—an effect comparable to that of the apoptosis-inducing chemotherapeutic agent DOX [65]. To assess the concentration-dependence of this inhibition, A549 cells were exposed to 12.5, 25, and 50 μM of JH62 for 24 h, which reduced viability by 20%, 48%, and 78%, respectively. After 32 h, viability decreased by 32%, 65%, and 84%, and after 48 h by 19%, 74%, and 92% (Figure 2B). Collectively, these data demonstrate that JH62 suppresses A549 cell proliferation in a time- and dose-dependent manner.

The cell colony formation assay, an in vitro method used to evaluate the proliferative capacity of cancer cells, was employed [66]. Compared to the control group, treatment with 6.3 and 12.5 μM JH62 significantly reduced the number of A549 lung cancer cell colonies by 34% and 71%, respectively (Figure 2C,D).

Selective targeting of cancer cells is a key characteristic of potential anticancer drugs. Enhancing the specificity toward cancer cells while maintaining broad-spectrum anticancer activity is an important objective in anticancer drug development [67]. To evaluate the spectrum and selectivity of JH62’s anticancer activity, we determined its half maximal inhibitory concentration (IC_50_) across a panel of non-cancer and cancer cell lines, using DOX as a reference chemotherapeutic agent (Figure 2E; Table 2). In non-cancer cells, JH62 exhibited relatively high IC_50_ values, including 47.2 ± 4.8 μM in MEF, 37.1 ± 0.7 μM in AML12, 44.1 ± 0.4 μM in WI-38, and 27.3 ± 4.9 μM in THLE-2, indicating low cytotoxicity toward non-cancer cells. In contrast, JH62 demonstrated significantly lower IC_50_ values against a broad-spectrum of human cancer cell lines, 17.2 ± 0.6 μM for HEPG2 (liver), 10.8 ± 0.4 μM for HEP3B (liver), 15.0 ± 0.4 μM for BxPC3 (pancreatic), 10.0 ± 0.2 μM for U87 (glioblastoma), 13.8 ± 3.3 μM for DU145 (prostate), 14.8 ± 0.1 μM for A549 (lung), 13.9 ± 1.3 μM for MCF7 (breast), 13.6 ± 2.7 μM for HeLa (cervical), and 8.6 ± 0.1 μM for HCT116 (colon), suggesting broad-spectrum anticancer activity.

As a comparison, DOX exhibited substantially lower IC_50_ values in both non-cancer and cancer cells. Specifically, IC_50_ values of DOX ranged from 1.2 to 5.1 μM in non-cancer cells (MEF, AML12, WI-38, and THLE-2) and from 0.7 to 3.9 μM across the tested cancer cell lines (Figure 2F; Table 2).

We next investigated potential synergistic interactions between JH62 and DOX, given their distinct mechanisms of action. A systematic combination study was performed by applying a concentration matrix based on their individual IC_50_ values to the same panel of cell lines. Drug interaction was quantified using the Zero Interaction Potency (ZIP) model on the SynergyFinder web tool (https://synergyfinder.fimm.fi/, accessed on 20 December 2025) (Table 2). In non-cancer cells (MEF, AML12, WI-38), the combination of JH62 and DOX primarily resulted in an additive effect (ZIP score < 10). An exception was noted in THLE-2 cells, which showed synergy (ZIP score > 10). Importantly, a clear synergistic effect was observed in the majority of cancer cell lines tested, with ZIP scores ranging from 10 to 20. The interaction remained additive only in HEPG2 liver cancer cells and MCF7 breast cancer cells (ZIP score < 10). These findings suggest that the combination of JH62 and DOX exerts a preferential synergistic effect against cancer cells.

To evaluate the efficacy of JH62 in inhibiting tumorigenesis in vivo, a cancer xenograft model was established by transplanting cancer cells into the right flank of nude mice. The mice were treated with JH62 (10 mg/kg body weight) twice weekly for three weeks, and tumor volume changes were regularly monitored during this period. External observations revealed that the tumor volume in the JH62-treated group was significantly smaller than that in the control group (Figure 2G and Figure S5). The tumor growth curve indicated that tumors in the control group continued to enlarge, whereas those in the JH62-treated group remained largely at the initial level (Figure 2I). After 20 days of treatment, the average weight of solid tumors in the JH62-treated group was 186.3 mg, compared to 693.4 mg in the control group. The average tumor size in the control group was approximately 900 mm^3^, while in the JH62-treated group, it was only about 190 mm^3^ (Figure 2H,I).

### 3.3. JH62 Induces Mitochondrial Dysfunction and Structural Disruption in A549 Lung Cancer Cells

**Figure 3:**
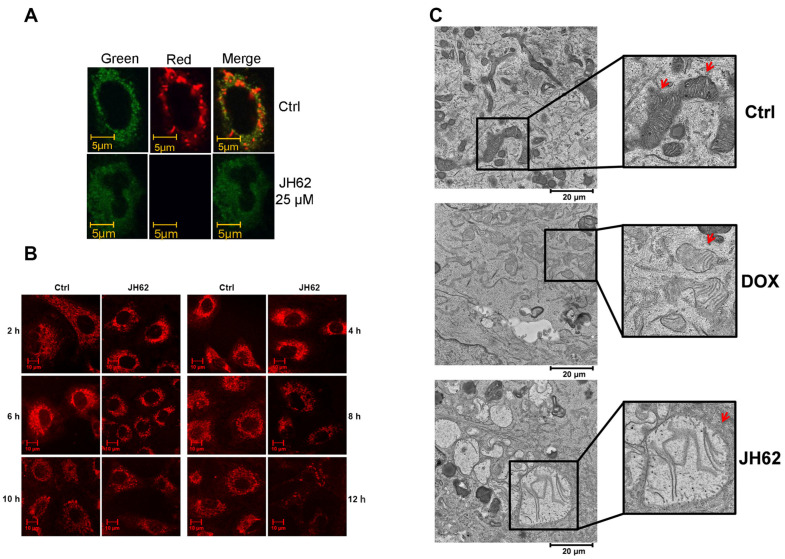
JH62 suppressed mitochondrial function. Mitochondrial JC-1 aggregate detection of mitochondria in JH62-treated A549 cells (**A**), green fluorescence indicates the monomeric form of the JC-1 dye, red fluorescence represents the aggregated form of JC-1, and the merged images show the overlay of the green and red fluorescence signals. MitoTracker Red confocal images (**B**) and transmission electron microscopic analysis (**C**) of mitochondria in JH62-treated A549 cells. Red arrows denote mitochondria. Ctrl, Dimethyl sulfoxide (DMSO) as solvent controls.

In cancer cells, mitochondria serve as central organelles for energy production and survival, critically influencing cell viability, immune evasion, tumor progression, and therapy resistance [68]. Given that JH62 inhibits A549 lung cancer cell growth, we next investigated its effects on mitochondrial integrity. The mitochondrial membrane potential (ΔΨm), resulting from the charge distribution across the inner mitochondrial membrane, is a key factor in maintaining normal mitochondrial function, and its collapse is a critical indicator of mitochondrial dysfunction [69]. We used the potential-sensitive fluorescent probe JC-1, which emits green fluorescence as a monomer at low concentrations and forms red-fluorescent aggregates in mitochondria with intact ΔΨm [70]. After treating A549 cells with JH62, the red fluorescence began to decrease within 2–3 h and reached the lowest level by 4 h, indicating that JH62 treatment disrupted the mitochondrial membrane potential (Figure 3A and Figure S6).

MitoTracker Red staining further revealed altered mitochondrial morphology and distribution following JH62 exposure. In JH62-treated cells, the MitoTracker Red signal transitioned from an initially focal, punctate, or circular pattern to a diffuse cytoplasmic distribution. Fluorescence intensity was significantly reduced, characteristic mitochondrial structures disappeared, and morphology became indistinct, suggesting that JH62 treatment led to the loss of mitochondrial membrane potential and disruption of structural integrity (Figure 3B).

Transmission electron microscopy (TEM) provided ultrastructural confirmation. In control cells, mitochondria exhibited intact morphology with clearly visible cristae. In contrast, mitochondria in JH62-treated cells commonly showed pathological alterations such as swelling, blurred inner membrane structures, and disrupted cristae (Figure 3C). These findings indicate that JH62 induces severe mitochondrial damage in lung cancer cells.

### 3.4. JH62 Induces Autophagic Cell Death Distinct from DOX-Mediated Apoptosis

**Figure 4:**
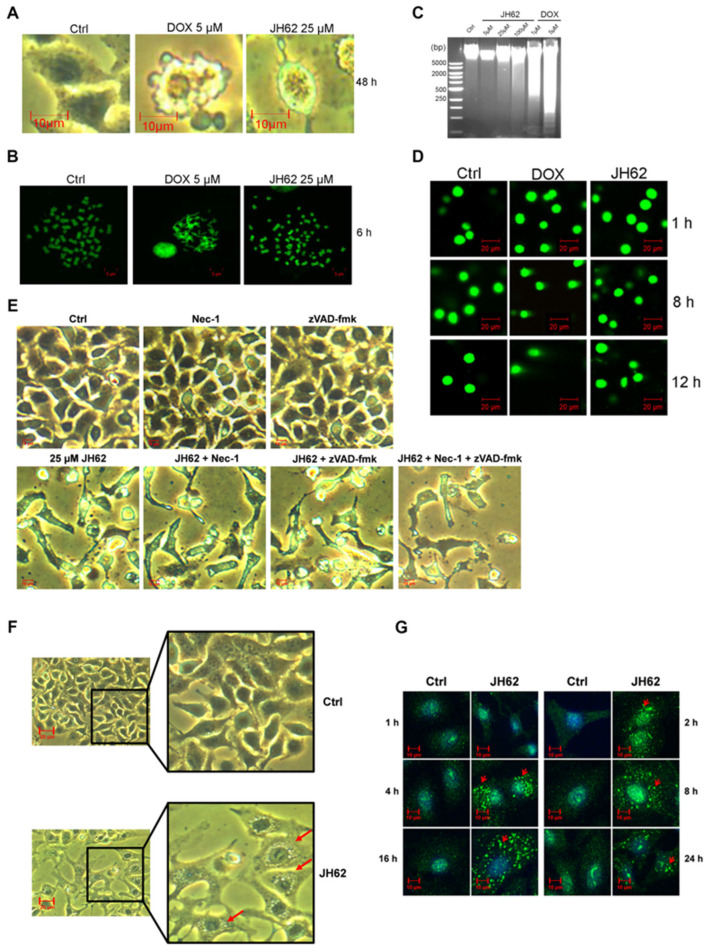
JH62 induces autophagy in A549 cells. (**A**) Phase contrast microscope images of DOX- or JH62-treated A549 cells. A549 cells were treated with 5 μM DOX or 25 μM JH62 for 48 h. (**B**) The chromosomes image of the DOX- or JH62-treated A549 cells. Before being stained and imaged, A549 cells were treated with 5 μM DOX or 25 μM JH62 for 6 h. (**C**) The genomic DNA electrophoresis assays of DOX- (1 and 5 μM) or JH62-treated (5, 25, and 100 μM) A549 cells. (**D**) Comet assay of DNA obtained from DOX- and JH62-treated A549 cells over 1, 8, and 12 h. (**E**) The apoptosis or necrosis inhibitor cannot block the A549 cell death induced by JH62. Nec-1, necrosis inhibitor. zVAD-fmk, apoptosis inhibitor. Phase contrast microscope images of A549 cells treated with DMSO (Ctrl), Nec-1, zVAD-fmk, JH62, JH62 + Nec-1, JH62 + zVAD-fmk, and JH62 + zVAD-fmk + Nec-1. (**F**) JH62 treatment induces autophagosome formation in A549 lung cancer cells. A549 lung cancer cells were treated with DMSO (Ctrl) or 25 μM JH62 for 24 h. Autophagosomes are indicated by red arrows. (**G**) Immunofluorescence analysis of LC3B in JH62-treated A549 cells. Cells were treated with JH62 for 1, 2, 4, 8, 16, and 24 h. Green fluorescence represents LC3B immunofluorescence signals, and red arrows denote LC3B punctate fluorescence signals. Ctrl represents the DMSO solvent control group.

DOX, a widely used chemotherapeutic agent, can trigger multiple forms of cell death including apoptosis, ferroptosis, and pyroptosis [71]. Although both JH62 and DOX induced death in A549 cells (Figure 1A), this study aimed to compare the modes of cell death induced by these two compounds. After 18 h of JH62 treatment, A549 cells became swollen, bubble-like structures emerged in the vicinity of the nucleus, and these vacuoles accumulated in the cytoplasm as the cytosol became transparent with prolonged exposure to JH62 (Figure 4A). In contrast, as previously reported [72], DOX-treated A549 cells showed a shrinking morphology, the formation of apoptotic bodies, and plasma membrane blebbing (Figure 4A).

A key mechanism of DOX is the induction of DNA damage [73]. The chromosome staining experiments showed that the chromosomes of DOX-treated A549 cells were fragmented and adhered together, whereas the chromosome of JH62-treated cells maintained normal morphology similar to the control (Figure 4B). The genomic DNA electrophoresis assays demonstrated that genomic DNA integrity remained unchanged even after 24 h of JH62 treatment (5–100 µM), whereas DOX treatment resulted in obvious DNA fragmentation (Figure 4C). Comet tail analysis further confirmed the maintenance of DNA integrity following JH62 treatment, while DOX-treated samples produced a distinct and typical comet tail (Figure 4D). The above findings demonstrate that JH62 treatment did not trigger the DOX-like DNA damage.

DOX can induce both apoptosis and necrosis in cancer cells, and the cytotoxic effects can be effectively suppressed by the apoptosis inhibitor zVAD-fmk and the necroptosis inhibitor Nec-1 [74]. Therefore, we investigated whether zVAD-fmk and Nec-1 could attenuate JH62-induced cell death in A549 lung cancer cells. As shown in Figure 4E, neither zVAD-fmk nor Nec-1, whether applied individually or in combination, could reverse the cytotoxicity of JH62 in A549 cells. These results indicate that the cell death triggered by JH62 is not associated with apoptosis or necroptosis.

We next investigated whether autophagy contributes to JH62-induced cell death. Microscopic observation revealed abundant translucent circular structures resembling autophagosomes in JH62-treated A549 cells, which were absent in the controls (Figure 4F). Immunofluorescence staining for LC3B, a core autophagy marker [75], showed that JH62 treatment progressively increased both the intensity and number of LC3B puncta—a hallmark of autophagosome formation—over 2–24 h, whereas control cells exhibited only a diffuse, weak LC3B signal (Figure 4G). These findings suggest that JH62 upregulates LC3B expression and promotes its punctate aggregation, supporting the conclusion that JH62 induces autophagic cell death in lung cancer cells.

### 3.5. The pqs Gene Cluster Mediates JH62 Biosynthesis in Pa

**Figure 5:**
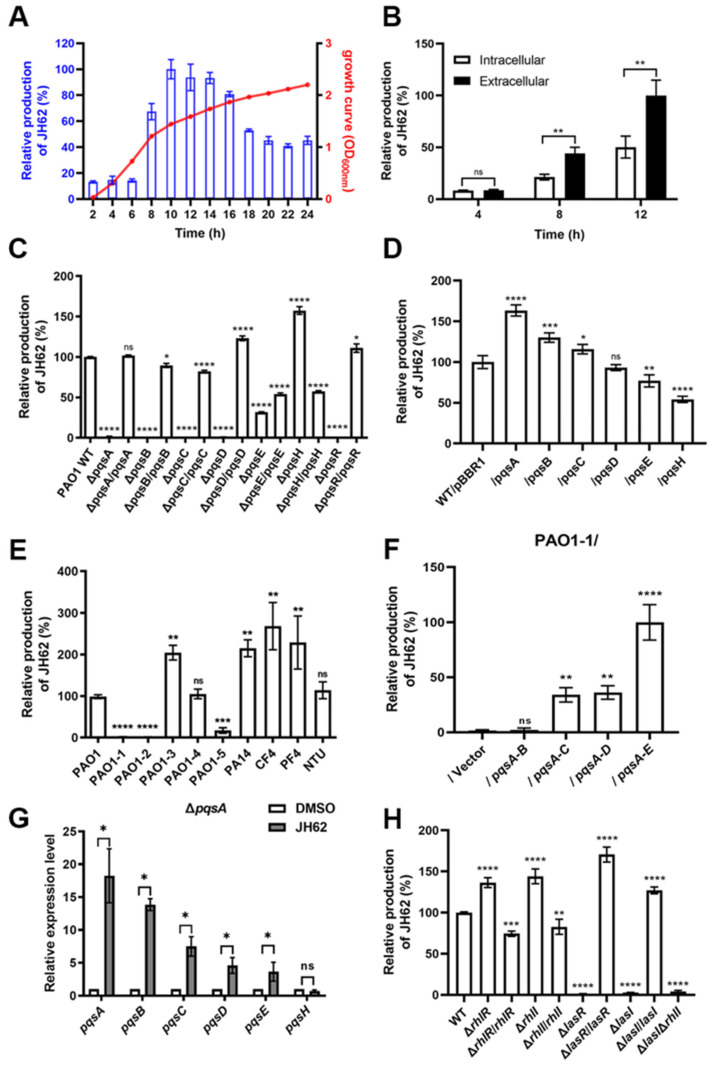
The *pqs* gene cluster in *Pa* mediates the biosynthesis of JH62. (**A**) The quantitative analysis of extracellular JH62 produced by *Pa* PAO1 at various growth periods. The blue column represents the extracellular production of JH62. The red curve represents the OD_600_ value of *Pa* culture. The maximum yield sample was defined as 100%. (**B**) The intracellular and extracellular production of JH62 in *Pa* PAO1 at 4, 8, and 12 h after incubation. The maximum yield sample was defined as 100%. (**C**) The production of JH62 in Δ*pqs* mutants and corresponding complemented strains. The yield of PAO1 WT was defined as 100%. (**D**) The analysis of rate-limiting enzyme in JH62 biosynthesis. WT/pBBR1, *Pa* PAO1 WT strain with empty pBBR1 plasmid./*pqs*, *Pa* PAO1 WT strain with corresponding *pqs* gene overexpressed pBBR1 plasmid. The yield of WT/pBBR1 was defined as 100%. (**E**) The production of JH62 in different *Pa* WT strains. The yield of PAO1 was defined as 100%. (**F**) The relative production of JH62 in PAO1-1 *pqs*-overexpressed strains compared to WT PAO1. pBBR1, empty vector as the negative control. The yield of WT PAO1 was defined as 100%. (**G**) Effect of exogenous JH62 on the transcriptional level of the *pqs* gene cluster in Δ*pqsA* mutant strain; data were analyzed by permutation test. ns, no significance; * *p* < 0.05. (**H**) The production of JH62 in *rhl* and *las* mutants and complemented strains. The yield of PAO1 WT was defined as 100%. Statistical analysis was performed using one way ANOVA. ns, no significance; * *p* < 0.05; ** *p* < 0.01; *** *p* < 0.001; **** *p* < 0.0001. *n* = 5. Error bars indicate standard deviations.

The biosynthetic pathway of JH62, a previously uncharacterized quinolone in *Pa*, was investigated. Time–course analysis by liquid chromatography-mass spectrometry (LC-MS) revealed that extracellular JH62 production correlated with bacterial density, peaking during the mid- to late-logarithmic growth phase (~10 h; Figure 5A). Furthermore, 66.5% of total JH62 was detected in the culture supernatant, with the remaining 33.5% associated with the cell pellet, indicating active secretion (Figure 5B).

*Pa* synthesizes over 50 structurally diverse quinolones via the *pqs* gene cluster (Figure S7A) [76]. Given the structural similarity of JH62 to known *pqs*-dependent quinolones such as HHQ and PQS (Figure S7B), in-frame deletion mutants of the *pqs* gene and the corresponding complemented strains were constructed. The results of quantitative experiments showed that JH62 production was abolished in Δ*pqsA*, Δ*pqsB*, Δ*pqsC*, Δ*pqsD*, and Δ*pqsE* mutants and restored upon complementation (Figure 5C). Among these, *pqsA* was identified as the rate-limiting step, as its overexpression significantly enhanced JH62 yield (Figure 5D). Furthermore, the production of JH62 exhibited differentiation across various *Pa* strains (Figure 5E). Notably, heterologous expression of the entire *pqsABCDE* cluster in the non-producing strain PAO1-1 resulted in JH62 levels twice those of wild-type (WT) PAO1 (Figure 5F).

Since the *pqs* gene cluster is responsible for JH62 biosynthesis, we determined whether JH62 itself could regulate the expression of this cluster, potentially establishing a feedback loop. To this end, we supplemented the JH62-deficient Δ*pqsA* mutant with exogenous JH62 and assessed the *pqs* cluster transcription. The results demonstrate that JH62 markedly upregulated the transcription of *pqsA*, *pqsB*, *pqsC*, *pqsD*, and *pqsE*, while leaving *pqsH* transcription unaffected, indicating a positive feedback loop that amplifies its production (Figure 5G).

Since JH62 accumulation is density-dependent and the *pqs* cluster is linked to QS, we evaluated the influence of two other QS systems, *las* and *rhl* [77], on JH62 production. The results showed that JH62 production was significantly decreased in Δ*lasI* and Δ*lasR*, and increased in Δ*rhlI* and Δ*rhlR* (Figure 5H). These results suggest that JH62 biosynthesis is integrated into the QS network and provide genetic strategies for enhancing its yield.

### 3.6. ADMET Evaluation Identifies JH62 as a Promising Lead Compound for Drug Development

Assessment of drug-like properties and ADMET (Absorption, Distribution, Metabolism, Excretion, and Toxicity) properties is essential for predicting in vivo pharmacokinetics and aiding clinical translation [78]. Accordingly, we conducted ADMET analysis on JH62 by using the online tools in websites SwissADME and ADMETlab 3.0. As summarized in Table 3, JH62 exhibits suitable physicochemical parameters such as low molecular weight (<500), topological surface area (<140 Å^2^), molar refractivity (<130), number of hydrogen bond donors (<5), and number of hydrogen bond acceptors (<10). The medicinal chemistry suggests that JH62 complies with Lipinski’s and “Golden Triangle” rules, supporting its drug-like properties. In addition, JH62 is predicted to be synthetically accessible, with a synthetic accessibility score well below 5. The ADMET evaluations revealed that JH62 exhibits favorable permeability, high bioavailability, and low toxicity. Structurally, JH62 did not trigger any PAINS (Pan Assay Interference Structures) alert. However, the Brenk method identified a structural alert related to the presence of an isolated alkene reactive group, which implies that JH62 has room for structural optimization. Collectively, these in silico data support JH62 as a promising and druggable lead candidate for anticancer development.

## 4. Discussion

Research on quinolone-type small molecules from *Pa* dates back to the 1950s [24]. Advances in detection and analytical techniques, particularly mass spectrometry-based metabolomic profiling, suggest that *Pa* may produce over 50 structurally diverse quinolone compounds, all sharing a 4-quinolone core [79]. In this study, we isolated a novel quinolone compound, JH62, from *Pa* and determined its structure as E-2-(tridec-4-en-1-yl)-quinolin-4(1H)-one (C_22_H_31_NO, molecular weight 325.48 Da). JH62 features a trans-4-tridecene side chain attached to the conserved quinolone core, representing a novel member of the *Pa*-derived quinolone family. The well-characterized quinolones produced by *Pa*, including HHQ and PQS, are established QS signal molecules that play crucial roles in regulating bacterial virulence, secondary metabolism, and interspecies interactions [80]. Structurally, JH62 shares the conserved quinolone core scaffold with these canonical QS molecules (Figure S7B), raising the possibility that it may interact with QS-related chemical frameworks. Indeed, beyond its anticancer properties, preliminary data indicate that JH62 also exhibits bioactivities related to QS and virulence regulation in *Pa*. Despite this shared scaffold, JH62 exhibits notable structural distinctions compared with previously characterized *Pa* quinolones. In particular, JH62 possesses an unusually long alkyl side chain comprising 13 carbon atoms, which represents the longest side chain reported to date among the experimentally isolated quinolone metabolites from this bacterium. Moreover, the position of the double bond within the alkyl chain differs from those of canonical QS molecules, further underscoring its structural uniqueness and expanding the known chemical diversity of *Pa*-derived quinolones. Earlier mass spectrometric analyses suggested the possible existence of C13-alkyl quinolones in *Pa* [81]; however, the isolation and structural characterization of JH62 provide direct experimental evidence for this quinolone subclass. The biosynthesis of all quinolone compounds in *Pa* depends on the multi-enzyme system encoded by the *pqs* gene cluster. The putative biosynthesis pathway of JH62 is shown in Figure S7C. In this context, JH62 represents a structurally distinct member of the bacterial quinolone family that links a QS-associated chemical scaffold with pronounced anticancer activity. While the present study focuses on its anticancer properties, a detailed investigation of potential QS-related or virulence-modulatory functions of JH62 will be addressed in future studies.

To contextualize the anticancer activity of JH62 within the JH62-type bacterial metabolites, we have noticed the growing reports on JH62-type compounds with similar bioactivity. Indeed, several quinolones produced by *Pa* itself possess anticancer properties. For instance, the well-known QS molecule PQS exhibits an IC_50_ of 19 μM against A549 cells, and both PQS and HHQ have been shown to impair mitochondrial function—a mechanism that aligns with our observations for JH62 [82,83]. Other *Pa*-derived quinolones also demonstrate anticancer effects, albeit often with lower potency (IC_50_ values typically ranging from 20 to 200 μM) [84]. Beyond *Pa*, quinolones from diverse bacterial genera show promising anticancer potential. Notable examples include 8-hydroxyquinoline from *Streptomyces* sp. (IC_50_ 26 μM against A549) [85], E-3-methyl-2-(2-octenyl)-4-quinolone from *Burkholderia* sp. (active against MCF7, HeLa, HT1080, and SiHa cells with IC_50_ values from 0.24 to 37 μM) [86], and E-1-hydroxy-2-(non-2-enyl)-3-methyl-4-quinolone from *Arthrobacter* sp. (IC_50_ 1.9 μM against HeLa) [87], and three quinolones from a sponge-associated *Pseudomonas* species (IC_50_ values of 33, 16, and 6 μM against KB cells) [88]. Clioquinol (5-chloro-7-iodo-8-hydroxyquinoline), which shares a structural similarity with JH62, also inhibits the growth of various cancer cell lines with IC_50_ values ranging from 6 to 43 μM [89]. This collective evidence underscores that the quinolone scaffold is a privileged structure for anticancer discovery [90], yet JH62 compares favorably with previously reported quinolone compounds, exhibiting low IC_50_ values (<20 μM) across a broad panel of cancer cell lines. Together with its structural uniqueness and selective cytotoxicity toward cancer cells, these observations further support the therapeutic potential of bacterial quinolones and positions JH62 as a particularly promising lead within this structural family.

The favorable safety profile of a drug candidate is paramount for successful clinical translation. In this study, JH62 exhibited high IC_50_ values (>25 μM) across murine (MEF and AML12) and human (WI-38 and THLE-2) non-cancer cell lines, indicating low cytotoxicity toward normal cells. In contrast, it demonstrated significantly greater potency (IC_50_ < 20 μM) against a diverse panel of cancer cell lines derived from lung, liver, breast, colon, and other tissues. This selectivity between normal and cancer cells—validated here in human models—suggests that JH62 can effectively target cancer cells while sparing normal ones, which is a highly desirable feature for anticancer agents aimed at minimizing off-target effects. For comparison, DOX showed consistently low IC_50_ values (ranging from 0.5 to 5 μM) against both non-cancer and cancer cells. The differential selectivity of JH62 highlights its potential for an improved therapeutic window compared to conventional chemotherapeutics like DOX. Furthermore, the broad-spectrum activity of JH62 against cancers of various origins strengthens its promise as a versatile lead compound for further development.

Previous work reported that *Pa* PAO1 can induce non-apoptotic cell death, though the mechanism remained unclear [91]. Our findings suggest that JH62-induced cell death is not apoptosis-associated and is accompanied by mitochondrial morphology and functional abnormalities, pointing toward a non-apoptotic death mechanism. It is important to note that the evidence supporting JH62-induced autophagic cell death in this study is primarily based on morphological observations (e.g., accumulation of autophagosome-like vesicles) and the punctate aggregation of the autophagy marker LC3B. While these findings strongly suggest the involvement of autophagy, further validation through pharmacological inhibition (e.g., using autophagy inhibitors) is warranted to conclusively establish the causal relationship between autophagy activation and JH62-mediated cytotoxicity. These experiments are currently underway. Establishing the precise role of autophagy will be a key focus of our ongoing research, which aims to delineate the complete molecular cascade triggered by JH62. Elucidating this pathway will not only solidify our understanding of JH62’s mechanism of action but may also reveal novel targets for combination therapies, particularly in cancers resistant to conventional apoptosis-inducing agents.

Pharmacokinetic profiling predicts the ADMET properties of a drug candidate [92]. JH62 possesses suitable physicochemical properties; its C13 alkyl side chain does not significantly impair its absorption, distribution, or permeability, indicating its promising potential as an anticancer lead compound. It is noteworthy that in silico prediction indicates a high PPB rate (~99%) for JH62, which could imply a low free drug concentration and potential limitations in pharmacological availability. However, a high PPB does not inherently preclude therapeutic efficacy. This is exemplified by numerous clinically successful drugs with a PPB exceeding 98%, such as warfarin and phenytoin, which remain effective in vivo. According to the free drug hypothesis and dynamic pharmacokinetic principles, a high PPB does not necessarily reduce the unbound drug concentration at the target site as it is often counterbalanced by corresponding adjustments in systemic clearance [93]. Critically, the potent activity of JH62 observed both in vitro and in vivo indicates that biologically effective concentrations can be achieved at the site of action.

The ADMET predictions presented in this study are derived from in silico analyses, which, while informative for early stage screening, necessitate experimental validation to fully characterize the compound’s pharmacokinetic and safety profile, as rightly noted. In light of this, our ongoing research is strategically focused on the structural optimization of the JH62 scaffold, aiming to enhance both its anticancer efficacy and druggability. We are conducting systematic structure–activity relationship (SAR) studies, exploring modifications such as the introduction of electron donating/withdrawing groups and variations in the alkyl side chain length. Encouragingly, preliminary results indicate that certain modifications, like the introduction of a methoxy group, reduce the predicted PPB to approximately 90% while increasing anticancer activity by approximately 41%. In subsequent studies, experimental evaluation of ADMET-related parameters, including hERG liability, microsomal stability, metabolic soft spots, and physicochemical properties such as solubility and logD, will be performed for JH62 and its optimized derivatives. Comprehensive experimental ADMET profiling and lead optimization will be addressed in future investigations to further support the development of JH62 as a promising anticancer lead compound.

Beyond its core anticancer activity, preliminary exploratory observations suggest that JH62 may influence STAT3-related signaling pathways, which raises the possibility that JH62 could exert additional effects related to inflammation or immune regulation. The STAT3 signaling axis represents a central regulatory node linking cancer development and inflammatory responses. However, the primary scope of the present work is the discovery and characterization of JH62 as a novel anticancer agent. Comprehensive evaluation of the anti-inflammatory potential and broader immunomodulatory effects of JH62 will be addressed in future investigations.

The discovery of JH62 provides evidence for the field of microbe-mediated anticancer therapy. Interkingdom interactions of microbiota in cancer therapy are therefore an important future direction, but the underlying mechanisms remain elusive and need extensive validation via preclinical models and clinical trials.

## 5. Conclusions

This study identified a novel and potent anticancer compound, JH62, from *Pa*. JH62 inhibited the proliferation and clonogenicity of lung cancer cells in a time- and dose-dependent manner, exhibited broad-spectrum anticancer activity with low cytotoxicity toward normal cells, and suppressed tumor growth in vivo. Treatment with JH62 induced mitochondrial morphological and functional disruption in cancer cells. The cell death triggered by JH62 is associated with autophagy. The biosynthesis of JH62 depends on the *pqs* gene cluster in *Pa*, and JH62 positively regulates the expression of this cluster. Together with favorable drug-like properties predicted by ADMET profiling, these findings establish JH62 as a promising lead compound for anticancer drug development.

## Figures and Tables

**Table 1:** The key metabolites of *Pa*.

Metabolite	Function	References
Pyocyanin	Oxidative stress; Host damage	[30]
Phenazines	Redox balance; Persistence	[31]
Rhamnolipids	Biofilm formation; Membrane disruption	[32]
Pyoverdine	Iron acquisition; Virulence regulation	[33]
Pyochelin	Iron acquisition	[34]
Hydrogen cyanide	Acute virulence; Respiratory inhibition	[35]
Acyl-homoserine lactone	Quorum sensing	[36]
Quinolones	Quorum sensing; Iron acquisition; Cytotoxicity	[37]
Alginate	Antibiotic tolerance; Chronic infection	[38]
Pel polysaccharide	Surface attachment; Cell aggregation	[39]
Psl polysaccharide	Biofilm initiation and maintenance	[40]
Acetate	Energy balance; Metabolic flexibility	[41]
Succinate	Preferred carbon source; Growth optimization	[42]
Glutamate	Growth and stress tolerance	[43]
Branched-chain amino acids	Environmental adaptability	[44]
Polyamine	Stress tolerance; Biofilm stability	[45]
Cyclic dipeptides	Quorum sensing; interspecies interactions	[46]
Lectins	Host cell attachment; Biofilm stability;	[47]
Volatile organic compounds	Virulence	[48]
Hemolysin	Cell lysis; Tissue damage; Virulence	[49]
Pyocins	Intraspecies competition; Niche dominance	[50]

**Table 2:** The IC_50_ value and ZIP Score of JH62 and DOX against different cell lines.

Cell Lines	JH62 IC_50_ (μM)	DOX IC_50_ (μM)	JH62/DOX ZIP Score
Non-cancer cell lines			
MEF	47.2 ± 4.8	3.9 ± 0.4	5.77 ± 2.17
AML12	37.1 ± 0.7	5.1 ± 1.3	5.83 ± 0.27
WI-38	44.1 ± 0.4	1.9 ± 0.3	4.91 ± 0.67
THLE-2	27.3 ± 4.9	1.2 ± 0.1	12.06 ± 1.79
Cancer cell lines			
HEPG2	17.2 ± 0.6	0.8 ± 0.1	8.31 ± 3.96
HEP3B	10.8 ± 0.4	3.3 ± 0.2	14.61 ± 1.18
BxPC3	15.0 ± 0.4	1.9 ± 0.1	13.79 ± 1.08
U87	10.0 ± 0.2	1.4 ± 1.0	13.85 ± 0.32
DU145	13.8 ± 3.3	1.9 ± 0.2	20.33 ± 6.66
A549	14.8 ± 0.1	2.1 ± 0.5	19.66 ± 5.28
MCF7	13.9 ± 1.3	1.2 ± 0.5	5.31 ± 3.23
HeLa	13.6 ± 2.7	0.7 ± 0.7	11.72 ± 3.18
HCT116	8.6 ± 0.1	3.9 ± 0.7	12.19 ± 2.30

Data are presented as the mean ± SD. IC_50_: Half maximal inhibitory concentration. ZIP Score ≤ 10: additive; ZIP Score > 10: synergistic.

**Table 3:** The drug-likeness and ADMET properties evaluation of JH62.

Properties	JH62
Physicochemical properties	
Molecular Weight	325.24
No. heavy atoms	24
No. aromatic heavy atom	10
No. rotatable bonds	11
No. H-bond donors	1
No. H-bond acceptors	1
Molar refractivity	106.74
TPSA	32.86 Å^2^
Medicinal chemistry	
PAINS	0
Brenk	1
Synthetic accessibility	3.14
Lipinski rule	Accepted
Golden triangle rule	Accepted
Absorption	
Intestinal permeability	−5.004 ●
MDCK permeability	−4.703 ●
PAMPA	0.003 ●
Human intestinal absorption	0.065 ●
Oral bioavailability	0.122 ●
Distribution	
BBB	0.0005 ●
VDss	0.5690 ●
PPB	99.0% ●
Metabolism	
CYP1A2 inhibitor	Inhibitor
CYP2C9 inhibitor	Inhibitor
CYP2D6 inhibitor	Inhibitor
CYP3A4 inhibitor	Inhibitor
CYP2B6 inhibitor	Inhibitor
Excretion	
Plasma clearance	4.937 ●
The half-life	0.352
Toxicity	
Drug-induced liver injury	0.179 ●
AMES Toxicity	0.132 ●
Rat oral acute toxicity	0.181 ●
Carcinogenicity	0.186 ●
Eye corrosion	0.014 ●
Drug-induced Neurotoxicity	0.258 ●
Genotoxicity	0.014 ●
RPMI-8226 Immunotoxicity	0.132 ●
Ototoxicity	0.342 ●

TPSA: Topological Polar Surface Area; PAINS: Pan Assay Interference Structures; MDCK: Madin–Darby Canine Kidney cells; PAMPA: The Parallel Artificial Membrane Permeability Assay; BBB: Blood–brain barrier penetration; VDss: Volume of distribution at steady state; PPB: Plasma protein binding. Colored label: The compound is evaluated as excellent (Green), medium (Yellow), or poor (Red) in the corresponding aspect.

## Data Availability

The original contributions presented in this study are included in the article/Supplementary Materials. Further inquiries can be directed to the corresponding authors.

## Supplementary Materials

The following supporting information can be downloaded at: https://www.mdpi.com/article/10.3390/microorganisms14010078/s1, Figure S1: HPLC separation and purification process of JH62; Figure S2: The full NMR spectrum of JH62; Figure S3: Comparative analysis of synthetic and purified JH62; Figure S4: Confocal microscope images of JH62-treated A549 cells; Figure S5: Tumor growth in xenograft mouse model with JH62 treatment; Figure S6: Mitochondrial JC-1 aggregate detection of mitochondria in JH62-treated A549 cells; Figure S7: Quinolone compounds biosynthesized by the *pqs* gene cluster of *Pa*.

## Abbreviations

The following abbreviations are used in this manuscript:

AQNO	2-alkyl-4-hydroxyquinoline N-oxide
DHQ	2, 4-dihydroxyquinoline
DMSO	Dimethyl sulfoxide
DOX	Doxorubicin
HHQ	2-heptyl-4-hydroxyquinoline
HNQ	2-nonyl-4-hydroxyquinoline
HPLC	High Performance Liquid Chromatography
HQNO	2-heptyl-4-hydroxyquinoline N-oxide
IC_50_	Half maximal inhibitory concentration
LC-MS	Liquid chromatography-mass spectrometry
NMR	Nuclear magnetic resonance
NQNO	2-nonyl-4-hydroxyquinoline N-oxide
*Pa*	*Pseudomonas aeruginosa*
PPB	Plasma protein binding
PQS	2-heptyl-3-hydroxy-4(1H)-quinolone
UQNO	2-undecyl-4-hydroxyquinoline N-oxide
WT	Wild type
