# Supplementary material for: A Novel Quinolone JH62 (E-2-(Tridec-4-en-1-yl)-quinolin-4(1H)-one) from Pseudomonas aeruginosa Exhibits Potent Anticancer Activity
Source: Microorganisms. 2025 Dec 30;14(1):78. doi: 10.3390/microorganisms14010078 (PMC12844237; doi:10.3390/microorganisms14010078)
Supplement: Supplementary file 1 [file microorganisms-14-00078-s001.zip › microorganisms-4031918-supplementary.pdf]

microorganisms

Supplementary Materials

A Novel Quinolone JH62 (E-2-(tridec-4-en-1-yl)-quinolin-4(1H)-one) from *Pseudomonas aeruginosa* Exhibits Potent Anticancer Activity

Qunyi Chen †, Jianhe Wang †, Xiaoyan Wu, Lantu Xiong, Lian-Hui Zhang * and Zi-Ning Cui *

Integrative Microbiology Research Centre, South China Agricultural University, Guangzhou 510642, China; chenqy@stu.scau.edu.cn (Q.C.); jhwang@scau.edu.cn (J.W.); wuxy@stu.scau.edu.cn (X.W.); xionglt0618@stu.scau.edu.cn (L.X.)

* Correspondence: ziningcui@scau.edu.cn (Z.N.C.); lhzhang01@scau.edu.cn (L.H.Z.); Tel.: +86-20-85288229 (Z.N.C.); +86-13570466460 (L.H.Z.)

† These authors contributed equally to this work.

Supplementary Materials includes:

Figures S1 to S7.

Academic Editor(s): Name

Received: date
Revised: date
Accepted: date
Published: date

Copyright: © 2025 by the authors. Submitted for possible open access publication under the terms and conditions of the Creative Commons Attribution (CC BY) license.

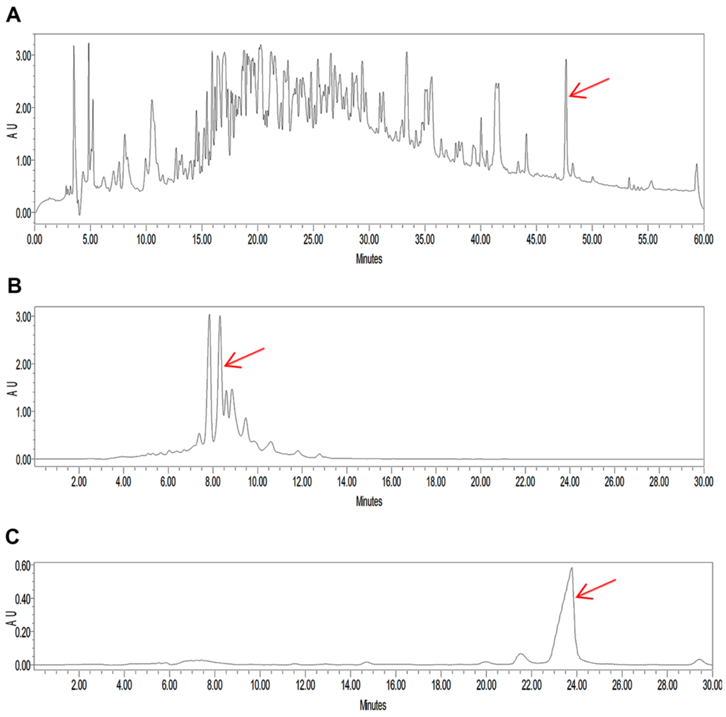

Figure S1. HPLC separation and purification process of JH62. **(A)**: Initial HPLC separation of the *Pa* supernatant extract using a 5-78% acetonitrile gradient; **(B)**: Further purification of the active fraction from (A) by HPLC with a 70% methanol isocratic elution; **(C)**: Subsequent purification of the active component from (B) by HPLC using a 40% methanol isocratic elution. Peaks corresponding to the components with anticancer activity are indicated by red arrows.

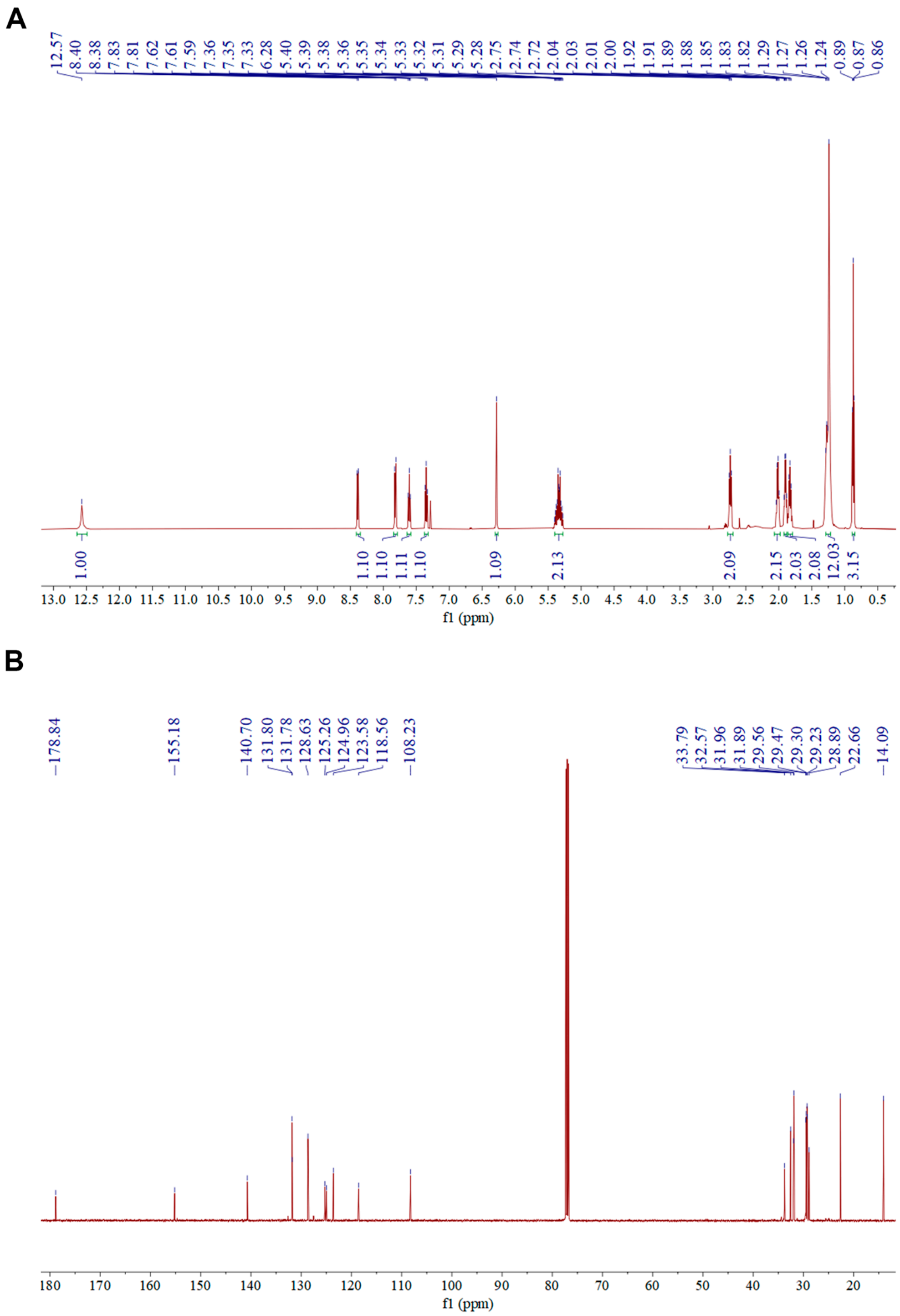

Figure S2. The full NMR spectrum of JH62. **(A)** The full ^{1}H NMR spectrum of the JH62. ^{1}H NMR (500 MHz, Chloroform-*d*) δ 12.57 (s, 1H), 8.39 (d, *J* = 9.6 Hz, 1H), 7.82 (d, *J* = 8.4 Hz, 1H), 7.61 (t, *J* = 8.5 Hz, 1H), 7.35 (t, *J* = 7.6 Hz, 1H), 6.28 (s, 1H), 5.40 – 5.28 (m, 2H), 2.74 (t, *J* = 7.8 Hz, 2H), 2.02 (q, *J* = 6.8 Hz, 2H), 1.90 (q, *J* = 6.4 Hz, 2H), 1.83 (t, *J* = 7.5 Hz, 2H), 1.26 (d, *J* = 16.6 Hz, 12H), 0.87 (t, *J* = 6.9 Hz, 3H); **(B)** The full ^{13}C NMR spectrum of the JH62. ^{13}C NMR (126 MHz, Chloroform-*d*) δ 178.84, 155.18, 140.70, 131.80, 131.78, 128.63, 125.26, 124.96, 123.58, 118.56, 108.23, 33.79, 32.57, 31.96, 31.89, 29.56, 29.47, 29.30, 29.23, 28.89, 22.66, 14.09.

https://doi.org/10.3390/microorganisms14010078

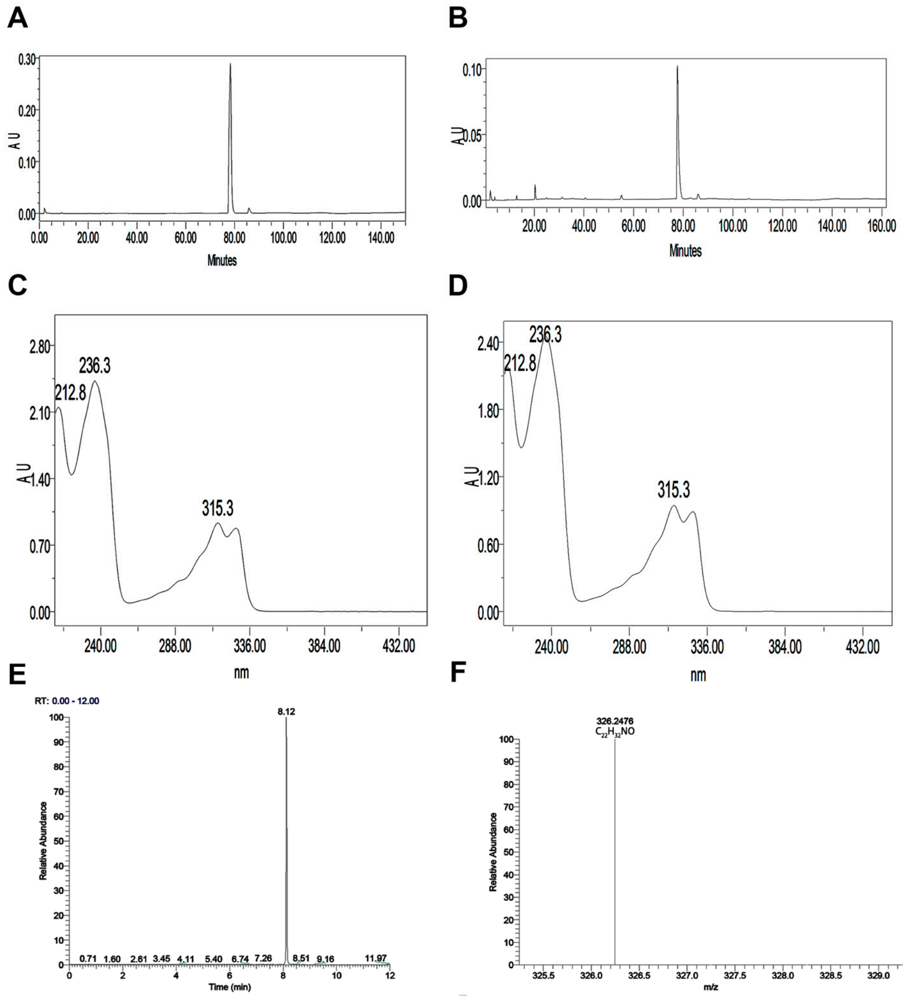

Figure S3. Comparative analysis of synthetic and purified JH62. HPLC profile **(A)** and UV spectrometric analysis **(C)** of synthesized JH62, respectively. HPLC profile **(B)** and UV spectrometric analysis **(D)** of purified JH62, respectively. **(E)** LC-MS chromatogram of chemically synthesized JH62. **(F)** Mass spectrum (from LC-MS analysis) of chemically synthesized JH62.

https://doi.org/10.3390/microorganisms14010078

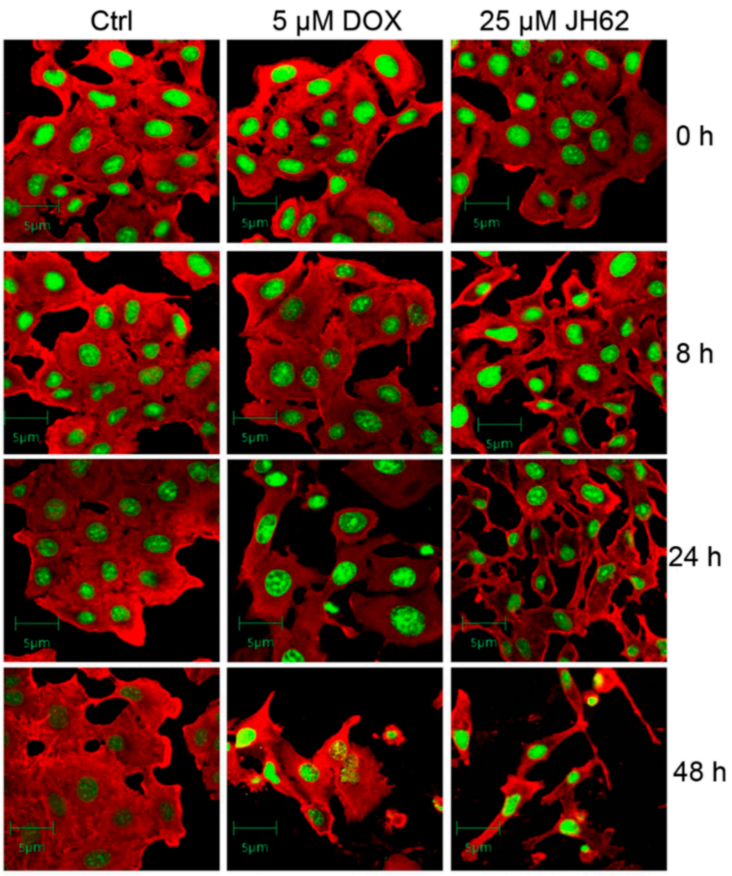

Figure S4. Confocal microscope images of JH62-treated A549 cells. The A549 cells were treated with 25 μM JH62 or 5 μM DOX for 0, 8, 24, and 48 h, respectively. Ctrl represented Dimethyl sulfoxide (DMSO) as solvent controls.

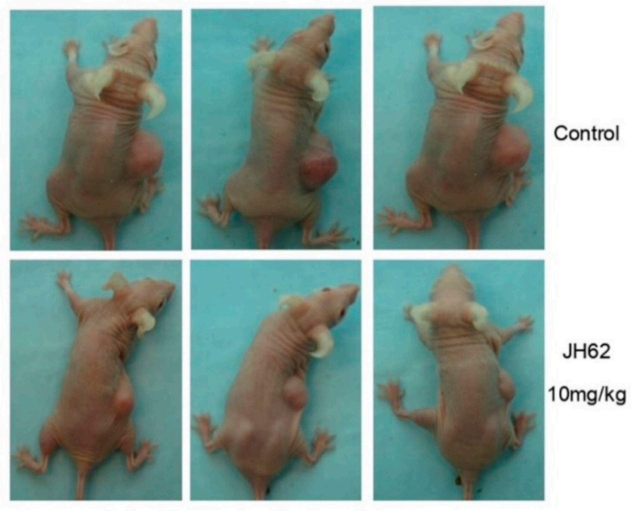

Figure S5. Tumor growth in xenograft mouse model with JH62 treatment. After being injected subcutaneously with cancer cells, BALB/c nude mice were treated via intraperitoneal injection with DMSO (control) or JH62 (10 mg/kg) twice a week for three weeks.

https://doi.org/10.3390/microorganisms14010078

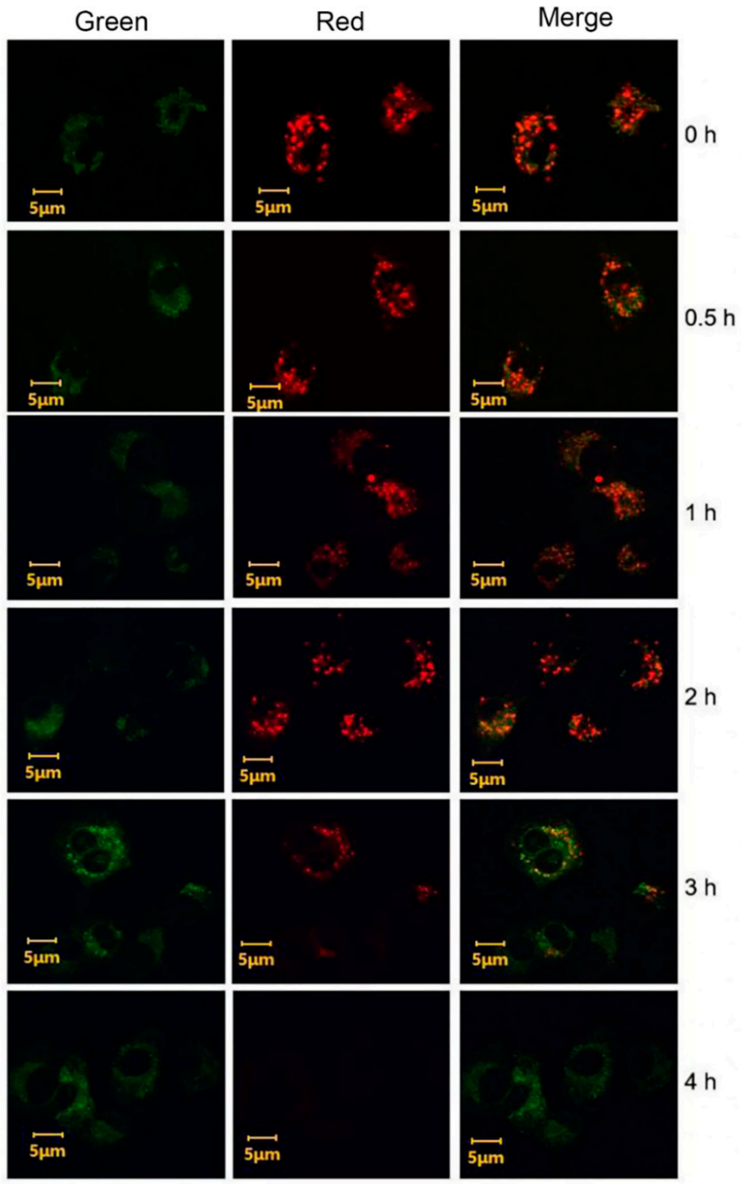

Figure S6. Mitochondrial JC-1 aggregate detection of mitochondria in JH62-treated A549 cells. The A549 cells were treated with 25 μM JH62 for 0.5, 1, 3, and 4 h. The stained cells were imaged by Zeiss LSM 510 META confocal microscope (Carl Zeiss, Jena, Germany) using × 40 lens and the 405, 488 and 561 nm lasers. Filters used: BP 505-550 for the green channel and BP 575-630 for the red channel.

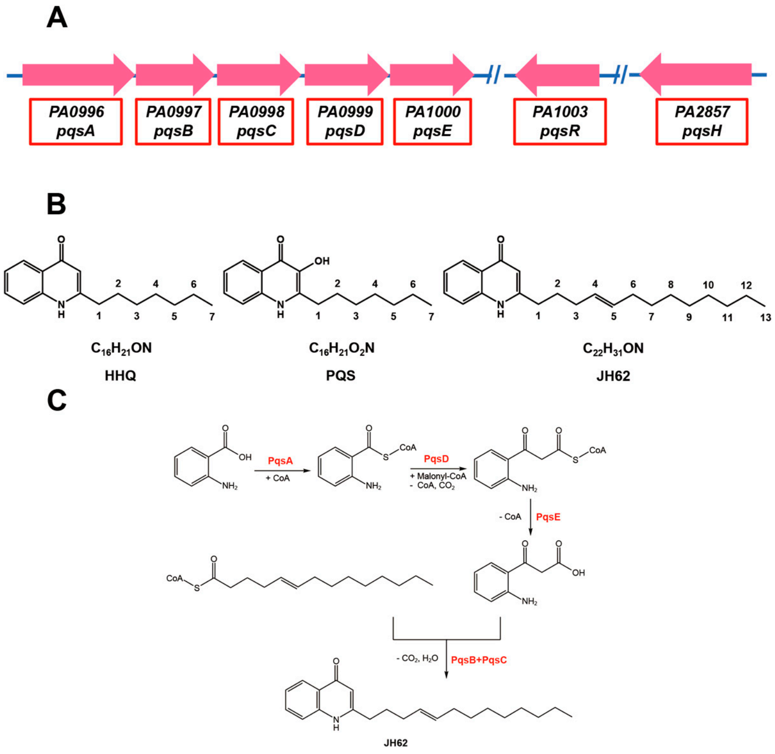

Figure S7. Quinolone compounds biosynthesized by the *pqs* gene cluster of *Pa*. **(A)** The genetic map of the *pqs* gene cluster. **(B)** The chemical structure of HHQ, PQS, and JH62. **(C)** The putative biosynthesis pathway of JH62.
